# Characteristics of In-Flight Medical Emergencies on a Commercial Airline in Mainland China: Retrospective Study

**DOI:** 10.2196/63557

**Published:** 2024-12-19

**Authors:** Ruizi Shi, Weisong Jiang, Jing Yang, Xiaomei Dong, Pei Yu, Shuai Zhou, Hanbing Shang, Wanying Xu, Er-zhen Chen, Zhitao Yang, Ying Zhou

**Affiliations:** 1 Shanghai Institute of Aviation Medicine, Ruijin Hospital, Shanghai Jiao Tong University School of Medicine, Shanghai, China; 2 Department of Emergency, Ruijin Hospital, Shanghai Jiao Tong University School of Medicine, Shanghai, China; 3 Aviation Medicine Branch of Shanghai Medical Association, Shanghai, China; 4 Department of Orthopedics, Ruijin Hospital, Shanghai Jiao Tong University School of Medicine, Shanghai, China; 5 Division of Medical Affairs, Ruijin Hospital, Shanghai Jiao Tong University School of Medicine, Shanghai, China; 6 Department of Neurosurgery, Ruijin Hospital, Shanghai Jiao Tong University School of Medicine, Shanghai, China; 7 Department of Neurosurgery, Ruijin-HaiNan Hospital, Shanghai Jiao Tong University School of Medicine, Hainan, China

**Keywords:** in-flight medical emergency, onboard emergency, aviation medicine, China, aircraft, medical emergency, aviation, airline, medical response, retrospective study

## Abstract

**Background:**

In-flight medical emergencies (IMEs) can have severe outcomes, including the deaths of passengers and aircraft diversions. Information is lacking regarding the incidence rate and characteristics of IMEs in most countries, especially in mainland China.

**Objective:**

The objective of this study was to investigate the incidence, patterns, and associated risk factors of IMEs in mainland China and to provide medical suggestions for the evaluation and management of IMEs.

**Methods:**

This population-based retrospective study examined electronic records for all IME reports between January 1, 2018, and December 31, 2022, from a major airline company in mainland China. Outcome variables included the medical category of the IMEs, the outcomes of first aid, and whether or not the IMEs led to a flight diversion. We calculated the incidence rate and death rate of IMEs based on the number of passengers and flights, respectively. A logistic regression model was used to investigate the factors associated with aircraft diversions.

**Results:**

A total of 199 IMEs and 24 deaths occurred among 447.2 million passengers, yielding an incidence rate of 0.44 (95% CI 0.39‐0.51) events per million passengers and 66.56 (95% CI 50.55‐86.04) events per million flights, and an all-cause mortality rate of 0.05 (95% CI 0.03‐0.07) events per million passengers and 7.50 (95% CI 4.81‐11.16) events per million flights. From 2018 to 2022, the highest incidence and mortality rates were observed in 2019 and 2020, respectively, while the lowest were in 2020 and 2021, respectively. Additionally, the highest incidence and mortality rates were observed between 6 PM to 6 AM and noon to 6 PM, respectively. There was a higher incidence rate of IMEs in the winter months. Moreover, the highest case-fatality rates were observed in 2019 (12/74, 16.2%), on flights traveling ≥4000 km (9/43, 20.9%), and on wide-body planes (10/52, 19.2%). Seizures (29/199, 14.6%), cardiac symptoms (25/199, 12.6%), and syncope or presyncope (19/199, 9.6%) were the most common medical problems and main reasons for aircraft diversion. The incidence of aircraft diversion was 42.50 (95% CI 37.02‐48.12) events per million flights. Narrow-body planes (odds ratio [OR] 5.69, 95% CI 1.05-30.90), flights ≥4000 km (OR 16.40, 95% CI 1.78‐151.29), and the months of December to February (OR 12.70, 95% CI 3.09‐52.23), as well as the months of March to May (OR 23.21, 95% CI 3.75‐143.43), were significantly associated with a higher risk of diversion.

**Conclusions:**

The occurrence of and deaths associated with IMEs are rare in mainland China, but a temporal trend shows higher incidence rates at night and in winter. The leading IMEs are cardiac symptoms, seizures, and syncope. The establishment of a unified reporting system for IMEs and ground-to-air medical support are of great value for reducing IMEs and deaths in the global community.

## Introduction

Air travel companies capture passengers’ favors through their convenience, safety, timeliness, reliability, and other airline products [1]. The number of commercial airline passengers traveling globally has been increasing and reached 4.46 billion in 2019 [2]. However, with an increasing number of annual passengers, increasing traveler age, and more long-haul flights, in-flight medical emergencies (IMEs) have become a serious problem that can be exacerbated by cabin environment; passengers’ physiological and psychological conditions; and other factors such as flight time, flight distance, and altitude of the airport [3-5].

IMEs can have severe outcomes, including the death of a passenger or an aircraft diversion. It has been reported that the pooled all-cause mortality rate was 0.21 (95% CI 0‐0.76) per million passengers [6]. Mostly, IMEs have been evaluated and managed by cabin personnel during flights [7-9]. However, the airplane cabin is not the optimal environment for patient resuscitation due to limited medical equipment and personnel, which can lead to an aircraft diversion to a suitable aerodrome by the pilot [10]. The most common reasons for an aircraft diversion are different among countries and include chest pain, epileptic seizures, and respiratory symptoms [9,11]. The 4 most common medical conditions during flights are syncope, gastrointestinal events, respiratory problems, and neurological problems [6], and the most worrisome are sudden cardiac arrest and death [12]. IMEs can differ among countries because they are usually associated with passengers’ demographics and underlying conditions, and data from previous studies have suggested that the demographics of air passengers can vary across countries [13-15]. However, information is lacking regarding the incidence rate and characteristics of IMEs in most countries, including China.

China is one of the largest domestic air travel markets in the world, accounting for 25% of global air passenger demand prior to the COVID-19 pandemic [16,17]. In mainland China, the total number of passengers carried on scheduled services rose to 659.93 million in 2019, which is 7.9% higher than the previous year, while the number of departures reached 4.96 million in 2019 [18]. China’s civil aviation sector has remained the world’s second-largest in terms of passenger trips for 15 years [19]. In terms of passenger demographics, there are differences between Chinese airlines and commercial airlines from other countries, with Chinese airlines exhibiting a higher male-to-female passenger ratio and adults aged 30‐45 years accounting for the largest proportion of travelers in China [20-22]. The Review of Standard Passenger Weights Final Report by the European Union Aviation Safety Agency shows that male passengers have a mean weight of 82.2 kg and female passengers have a mean weight of 67.5 kg, while the 2020 Report on Chinese Residents’ Chronic Diseases and Nutrition indicates that the average weight for Chinese men is 69.6 kg [23]. Moreover, the World Obesity Atlas 2023 published by the World Obesity Federation also showed that the prevalence of obesity among Chinese adults is much lower than most North American and European countries [24]. Therefore, the occurrence of IMEs may differ among countries due to the disparities in demographic and physical characteristics among travelers from China and other countries. However, there is limited knowledge about the occurrence of IMEs for commercial airlines in mainland China. Therefore, an exploratory study was conducted to investigate the frequency and types of IMEs.

The specific study goals included (1) to identify the incidence of IMEs that occurred in mainland China; (2) to characterize IMEs, identifying the patterns and severity of these events; (3) to investigate the risk factors associated with in-flight deaths and aircraft diversion; and (4) to provide medical suggestions for the evaluation and management of IMEs.

## Methods

### Study Design and Setting

This was a retrospective study of a major airline company (the airline company name is not specified for privacy reasons), with more than 89 million annual passengers between January 1, 2018, and December 31, 2022. This commercial airline is one of the three largest airlines in mainland China, and its airline transportation turnover volume accounts for a fifth of mainland China’s total volume [25]. Electronic records, mainly consisting of free-form narrative summaries of IMEs, were acquired from this airline company. Notably, onboard events that occurred before takeoff or after landing were also included, such as during taxiing, a safety check before takeoff, and preparation for disembarkation.

### Outcome Measures

Outcome variables collected included the medical category of the IME, outcomes of first aid, and whether or not the medical event caused an unscheduled landing (flight diversion). Regarding the medical category, two doctors independently reviewed the summary of complaints or symptoms of the passenger and made a primary clinical diagnosis. Cases of uncertain classification were settled by a third doctor and resolved by discussion. In cases where information was lacking or a determination was impossible, the classification was listed as “unspecified.” Then, medical categories according to the organ system affected and nature of the illness were created. All events categorized as “other” were those with substantially lower incidences.

### Statistical Analysis

The data were presented as numbers and percentages for all categorical variables. To compare the groups, χ^2^ tests were performed. The frequency of each IME category, aircraft diversions, transportation to the hospital, and deaths were investigated. To calculate incidence rates and mortality rates for commercial aircraft passengers, the total number of reported IMEs and deaths from 2018 to 2022 were divided by the number of airline passengers or flights, respectively, and the 95% CIs were calculated. The statistics on the overall number of passengers and flights were obtained from both the airline company and the website of the Ministry of Transport of the People’s Republic of China [25].

To explore the factors associated with aircraft diversion, multivariate analyses were performed using logistic regression models. Variables with statistical significance in the univariate analysis were included in the multivariate analysis under the consideration of sample size and the frequency of events. Odds ratios (ORs) and 95% CIs were calculated. All reported *P* values are 2-sided, and a *P*≤.05 was considered statistically significant. All statistical analysis was performed using Stata 15.0 (Stata Corporation). Figures were also constructed using Prism 8.0 (GraphPad Software).

### Ethical Considerations

This study was approved by the Institutional Review Board of Ruijin Hospital (No. 2023325). The data for this investigation were event records received from a commercial airline. The raw data did not include personally identifying information like names, ID cards, or addresses. This study did not need informed consent since it involved the analysis of existing, anonymized data without direct human subject involvement. Individuals were not directly involved, hence, participation pay was not relevant. Furthermore, no photos or additional materials in the publication include identifying information; therefore, approval for image usage was unnecessary.

## Results

### Characteristics of IMEs

**Figure 1.**
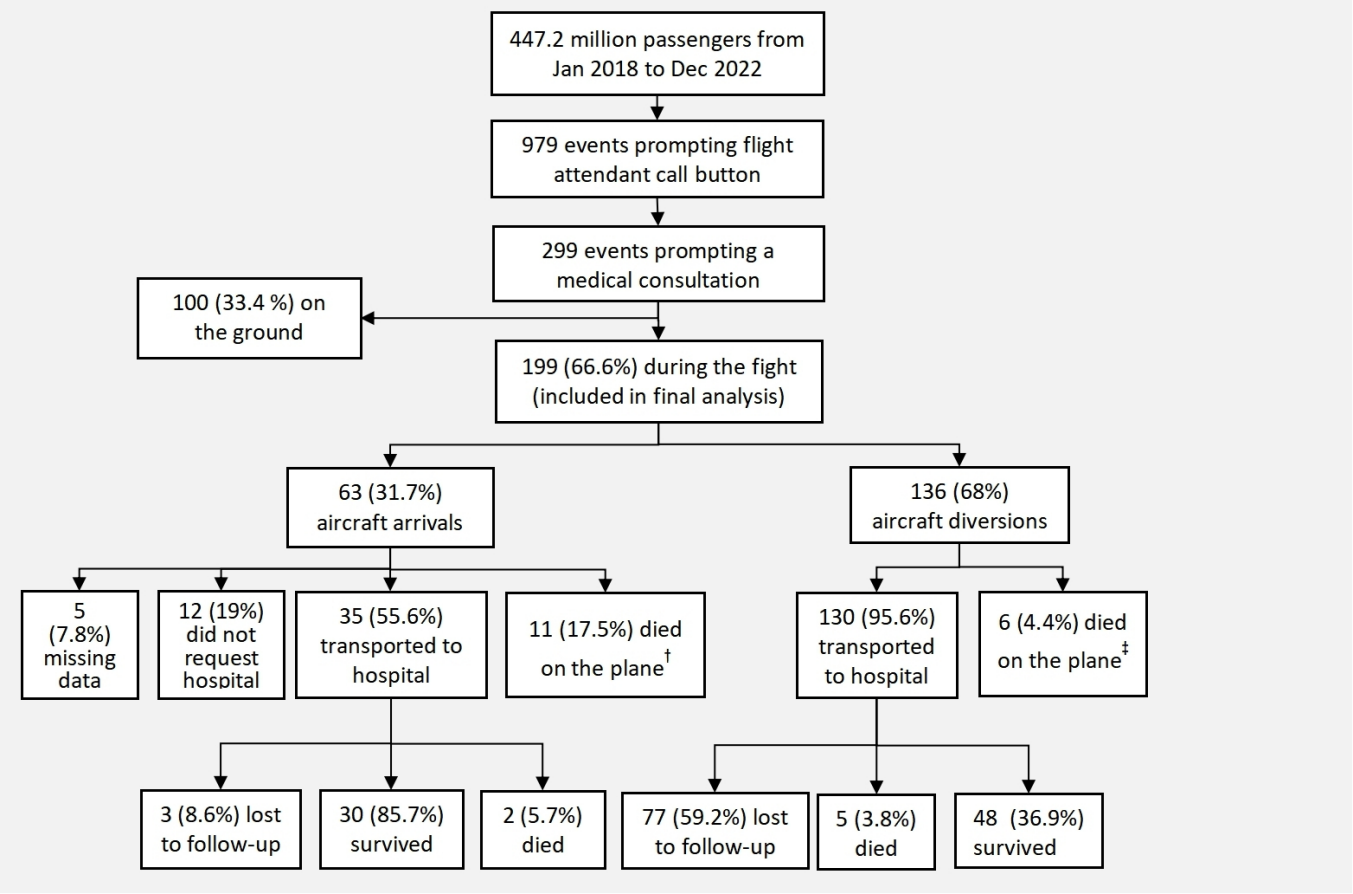
Outcomes of in-flight medical emergencies. Postflight follow-up data were not available from incomplete records of an in-flight medical emergency. ^†^Passengers who died on the plane when the aircraft was near the destination or in cases diagnosed as cardiopulmonary arrest (n=8), cachexia (n=1), or sudden syncope (n=2), in which cardiopulmonary resuscitation (CPR) could not help and after which family members of these cases (if present) indicated to stop CPR, resulting in the flight arriving at the scheduled destination. ^‡^Passengers who died on the plane were given CPR with the assistance of medical volunteers but attempts were unsuccessful.

Between 2018 and 2022, 447.2 million passengers were carried by 3.2 million flights offered by the airline of interest, with the occurrence of 979 medical events identified by flight attendants, among which 299 required further medical support after the evaluation by crew members. After excluding 100 events that occurred on the ground, 199 IMEs were included in the final analysis (Figure 1).

The age of the passengers among the 199 IMEs ranged from 2 months old to 93 years old, and females constituted 35.7% (71/199) of IMEs (Table 1). Of these IME records, all onboard assistance was provided by health care professionals, accounting for 26.1% (52/199) of all events. The use of automated external defibrillators was relatively rare (6/18, 33.3%) for cardiopulmonary arrest cases in this study. An aircraft diversion occurred in 68.3% (136/199) of cases, yielding an incidence of 42.50 (95% CI 37.02‐48.12) events per million flights from 2018 to 2022. The characteristics of passengers with different flight outcomes were significantly different. Passengers with IMEs that resulted in an aircraft diversion were more likely to be in the unknown age group (51/136, 37.5%), be on narrow-body planes (115/136, 84.6%), go without volunteer medical assistance (109/136, 80.2%), and have an unknown medical history (75/136, 55.1%; Table 1).

**Table 1.** Characteristics of in-flight emergencies by flight outcome.

Categories	All events (n=199), n (%)	Flight outcomes		*P* value
		Aircraft diversion (n=136), n (%)	Scheduled destination (n=63), n (%)	
Age groups (years)				.01
<18	13 (6.5)	10 (7.3)	3 (4.8)	
18‐34	35 (17.6)	24 (16.7)	11 (17.5)	
35‐49	24 (12.1)	16 (11.8)	8 (12.7)	
50‐64	41 (20.6)	26 (19.1)	15 (23.8)	
≥65	23 (11.6)	9 (6.6)	14 (22.2)	
Unspecified	63 (31.7)	51(37.5)	12 (19)	
Sex				.001
Female	71 (35.7)	40 (29.4)	31 (49.2)	
Male	82 (41.2)	55 (40.4)	27 (42.9)	
Unspecified	46 (23.1)	41 (30.2)	5 (7.9)	
Type of aircraft				<.001
Wide-body plane	52 (26.1)	21 (15.4)	31 (49.2)	
Narrow-body plane	147 (73.9)	115 (84.6)	32 (50.8)	
Flight distance (km)				<.001
<1000	16 (8)	9 (6.6)	7 (11.1)	
1000‐1999	91 (45.7)	73 (53.7)	18 (28.6)	
2000‐3999	49 (24.6)	37 (27.2)	12 (19)	
≥4000	43 (21.6)	17 (12.5)	26 (41.3)	
Volunteer provider of medical assistance				<.001
No	147 (73.9)	109 (80.2)	38 (60.3)	
Yes	52 (26.1)	27 (19.8)	25 (39.7)	
Death				<.001
No	95 (47.7)	48 (35.3)	47 (74.6)	
Yes	24 (12.1)	11 (8.1)	13 (20.6)	
Unspecified	80 (40.2)	77 (56.6)	3 (4.8)	
AED^a^ use^b^				.17
No	12 (66.7)	6 (85.7)	6 (54.6)	
Yes	6 (33.3)	1 (14.3)	5 (45.4)	
Medical history				.04
No	42 (21.1)	27 (19.9)	15 (23.8)	
Yes	61 (30.7)	34 (25)	27 (42.9)	
Unknown	96 (48.2)	75 (55.1)	21 (33.3)	
Season				.003
Mar-May	46 (23.1)	36 (26.5)	10 (15.9)	
Jun-Aug	32 (16.1)	21 (15.4)	11 (17.5)	
Sep-Nov	65 (32.7)	34 (25)	31 (49.2)	
Dec-Feb	56 (28.1)	45 (33.1)	11 (17.5)	

a AED: automated external defibrillator.

b Only calculated among cardiopulmonary arrest cases (n=18).

### Medical Problems and Outcomes of IMEs

The most common medical problems were seizures (29/199, 14.6%), cardiac symptoms (25/199, 12.6%), and syncope or presyncope (19/199, 9.6%). The leading cause of death was cardiopulmonary arrest (16/24, 66.7%), with a case-fatality rate of 88.9% (16/18). The most common IMEs resulting in an aircraft diversion were cardiac symptoms (23/136, 16.9%), seizures (20/136, 14.7%), and syncope or presyncope (13/136, 9.5%) (Table 2). According to the categories of medical problems, we made medical suggestions for passengers, aircraft crew, and medical volunteers on how to prevent and deal with IMEs (Figure S1 in Multimedia Appendix 1).

**Table 2.** In-flight medical emergencies according to medical problem and outcomes.

Categories	All events (n=199), n (%)	Flight outcomes		Case-fatality rate^a^, n/N (%)
		Aircraft diversion (n=136), n (%)	Scheduled destination (n=63), n (%)	
Cardiac symptoms	25 (12.6)	23 (16.9)	2 (3)	1/25 (4)
Seizures	29 (14.6)	20 (14.7)	9 (14)	0/29 (0)
Syncope or presyncope	19 (9.6)	13 (9.5)	6 (10)	1/19 (5)
Abdominal pain	10 (5)	6 (4.4)	4 (6)	0/10 (0)
Respiratory symptoms	14 (7)	8 (5.9)	6 (10)	1/14 (7)
Cardiopulmonary arrest	18 (9.1)	7 (5.1)	11 (18)	16/18 (89)
Hysteria	11 (5.5)	8 (5.9)	3 (5)	0/11 (0)
Nausea or vomiting	9 (4.5)	6 (4.4)	3 (5)	2/9 (22)
Agitation or psychiatric symptoms	2 (1)	1 (0.7)	1 (2)	0/2 (0)
Other neurologic symptoms	6 (3)	5 (3.7)	1 (2)	2/6 (33)
Infectious disease	4 (2)	3 (2.2)	1 (2)	0/4 (0)
Hypotension	4 (2)	0 (0)	4 (6)	0/4 (0)
Hypoglycemia	8 (4)	6 (4.4)	2 (3)	1/8 (12)
Allergic reaction	8 (4)	4 (2.9)	4 (6)	0/8 (0)
Obstetrical or gynecologic symptoms	2 (1)	2 (1.5)	0 (0)	0/2 (0)
Other	16 (7.4)	10 (7.3)	6 (10)	0/16 (0)
Unspecified	14 (7)	14 (10.3)	0 (0)	0/14 (0)

a There were 24 deaths across all 199 in-flight medical emergencies.

### Incidence and Mortality Rates of IMEs

The incidence rate of IMEs was 0.44 (95% CI 0.39‐0.51) events per million passengers and 66.56 (95% CI 50.55‐86.04) events per million flights. The all-cause mortality rate of IMEs was 0.05 (95% CI 0.03‐0.07) events per million passengers and 7.50 (95% CI 4.81‐11.16) events per million flights. From 2018 to 2022, the highest number of IMEs by passengers was observed in 2019 (0.60, 95% CI 0.51‐0.69, events per million), while the lowest was observed in 2020 (0.29 events per million passengers). As for the death rates of IMEs, the highest rate occurred in 2019, with 0.10 (95% CI 0.05‐0.16) events per million passengers, and the lowest occurred in 2020 (0.01, 95% CI 0.00‐0.07, events per million).

**Figure 2.**
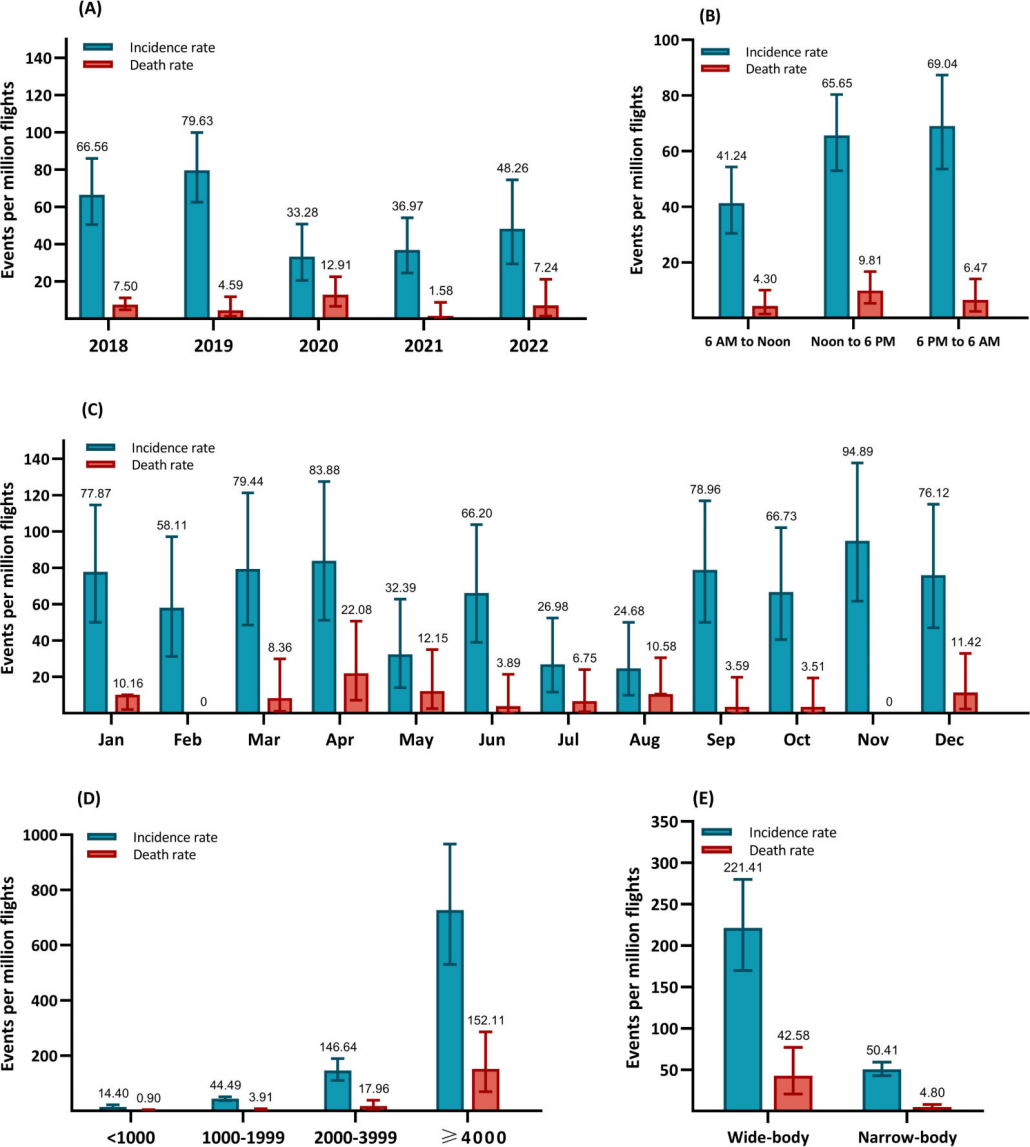
Incidence and mortality rates of in-flight medical emergencies by calendar year (A), time period (B), month (C), flight distance in kilometers (D), and aircraft type (E).

Figure 2 shows the incidence rates and death rates of IMEs per million flights by flight information. From 2018 to 2022, the highest incidence of IMEs was 79.63 (95% CI 62.53‐99.95) events per million flights in 2019, while the highest death rate for IMEs was 12.91 (95% CI 6.67‐22.55) events per million flights in 2020. The lowest incidence rate of IMEs was observed in 2020 (33.28, 95% CI 20.60‐50.87, events per million), while the lowest death rate was observed in 2021 (1.58, 95% CI 0.00‐8.83, events per million; Figure 2A). For the time period the IMEs occurred, the highest incidence and mortality rates were observed at 6 PM to 6 AM and noon to 6 PM, respectively (Figure 2B). The highest incident rates of IMEs were observed in November and April, while the highest mortality rate was observed in April (Figure 2C). Moreover, the highest incidence and mortality rates of IMEs were both observed in flights ≥4000 km long (Figure 2D). As for the type of aircraft, a higher incidence rate and mortality rate were observed in wide-body planes, despite most events occurring in narrow-body planes (Figure 2E).

### Case-Fatality Rate of IMEs

Of the 199 IMEs recorded, the highest case-fatality rates were observed in 2019 (12/74, 16.2%), on flights traveling ≥4000 km (9/43, 20.9%), and on wide-body planes (10/52, 19.2%). Regarding the other flight characteristics, higher case-fatality rates occurred in April (5/19, 26.3%), May (3/8, 37.5%), July (2/8, 25.0%), and August (3/7, 42.9%) (Table S1 in Multimedia Appendix 1).

### Factors Associated With IMEs and IME-Related Deaths

Table S2 in Multimedia Appendix 1 shows the results of the multivariable analysis. Compared with wide-body planes, narrow-body planes had a 5.69 (95% CI 1.05‐30.90) times higher risk of an aircraft diversion. Flights traveling a distance of ≥4000 km had a higher risk of diversion compared with flights traveling a distance of <1000 km (OR 16.40, 95% CI 1.78‐151.29). Moreover, the risk of an aircraft diversion was higher during the months of December to February (OR 12.70, 95% CI 3.09‐52.23) and the months of March to May (OR 23.21, 95% CI 3.75‐143.43) than the months of September to November.

## Discussion

### Principal Findings

In this study, we found that the incidence rate of IMEs was 0.44 events per million passengers, the all-cause mortality rate was 0.05 per million passengers, and the incidence rate of aircraft diversions was 42.5 per million flights during the study period of 2018 to 2022. Cardiac symptoms, seizures, and syncope or presyncope were the three leading IMEs and the main causes of aircraft diversions.

Our study indicates the incidence of IMEs in mainland China was substantially lower compared to other countries. In a review of IMEs, the incidence rate ranged between 24 and 130 IMEs per million passengers [8], while it was 0.44 per million passengers in our study. There might be some possible reasons for the lower incidence of IMEs in mainland China. First, the definition of an IME differs depending on the country. An IME, defined by the Civil Aviation Administration of China, includes diseases or deaths resulting in abnormal operations of an aircraft, such as an aircraft diversion; sudden diseases or deaths during airline flights; and public health emergencies [26,27]. The term IME in other countries refers to all IMEs, including nonserious and serious medical problems [27,28]. The failure of the global community to agree on the definition of an IME might slow progress in the prevention and management of IMEs. We recommended a stricter definition only including serious medical events, as our study indicated that most IMEs are self-limiting or are effectively evaluated and treated, which would not affect the health of the passengers or flight routes. During these flights, severe illness was infrequent and death was rare.

Second, variations in reported IME frequencies may be related to reporting behaviors. Each airline tracks its own medical events, making it difficult to determine the true incidence of specific illnesses during a flight. Also, there is no central registry of IMEs in the global community. Our study demonstrated that a substantial proportion of patients who had IMEs have been lost to follow-up without providing a complete set of information. Specifically, information associated with the deaths (ie, on the aircraft, in transport to the hospital, in the hospital) can remain unknown, which makes it difficult to manage and reduce deaths from IMEs. Therefore, a reporting form for IMEs was designed, with the advantages of being simple and comprehensive (Table S3 in Multimedia Appendix 1). The information gained using the recommended reporting form for IMEs would be of benefit to airlines, aerospace medical researchers, and the traveling public. A globally unified reporting system could provide consistent global data and facilitate the timely analysis of IMEs. These data could help identify high-risk groups vulnerable to IMEs, provide insights for first aid kits and wearable devices on board, and improve decision-making for aircraft diversions. These systems could prevent IMEs; provide effective treatment for IMEs; reduce the deaths of passengers; and decrease health, economic, environmental, and social costs.

In this study, we found that cardiac symptoms, seizures, and syncope or presyncope were the most common IMEs. In the United States, the most common medical events are syncope, respiratory symptoms, and nausea or vomiting [29], and in Turkey, they are headache, dizziness, and syncope or presyncope [9]. Studies from different countries have shown common symptoms [3,9,28,29]. Understanding which illnesses are most likely to occur would improve emergency medical assistance, including the prevention and management of IMEs for commercial flights in mainland China. Knowing which patients are most likely to become ill can help the aviation medicine community develop training materials for physicians who might be asked to volunteer. Therefore, we made medical suggestions for passengers, aircraft crew, and medical volunteers on how to prevent and deal with IMEs.

Prevention of IMEs is the best way to address them. The passengers’ symptoms can often be managed in collaboration with flight attendants and health care professionals. When the need for evaluation or intervention exceeds their capabilities, flight attendants may seek ground-to-air help via telemedicine. In cases of IMEs with complicated symptoms, management by onboard health care professionals or professional ground-to-air medical advice is recommended [30]. Notably, due to the incomplete records during IME reporting, a considerable portion of the incidents could not have their causes determined and were categorized as unspecified. Additionally, many cases that lead to an aircraft diversion and a transfer to a hospital are not followed up, leading to a lack of known health outcomes for these passengers.

In this study, we observed that the incidence of IMEs was highest in 2019 and lowest in 2020, which might be attributed to the reduction in the number of flights during the COVID-19 pandemic. Moreover, we have found that IMEs occurred more frequently at night than any other time, and there was a higher incidence rate in the winter months. Some research has shown that certain diseases tend to have higher incidence rates at night and in the winter months, which might partially explain the results of this study [31-34]. The population of air travelers has transitioned to a demographic that is older with more comorbidities [35,36], making the occurrence and management of IMEs a potentially greater challenge. Airlines need to make specific medical preparations to reduce medical events during these time periods. Our study also found that long-distance flights (≥4000 km) had a 16-fold higher risk of diversion compared to flights traveling <1000 kms, which was consistent with a previous finding suggesting that approximately 68.8% of IMEs occurred on intercontinental flights [37]. Therefore, the enhanced emergency medical kit we have recommended in Table S4 in Multimedia Appendix 1 should be taken into consideration by airline companies to increase treatment options.

Having an onboard doctor might benefit the passengers and cabin crew. However, in our study, the participation rate of medical volunteers was about 20%, which is lower than rates observed in Turkey and the United States [12,29]. A study on ground-to-air emergency calls reported a participation rate of 65% for medical volunteers in Asia in 2006 [38]. Many physicians are not trained in aviation medicine and do not know the effects of cabin environments on passenger health, how to overcome IMEs, their legal responsibilities for onboard patient care, and the medical resources available to them on board [38-41]. This situation may result in improper medical treatment and an unnecessary aircraft diversion [8,42]. Therefore, airline companies should not rely on having health care volunteers on board. Instead, they can provide in-house ground-to-air medical support or use commercial companies that provide immediate access to a remote doctor [43]. Enhanced ground-to-air telemedicine could reduce aircraft diversions by 70% [44]. Also, aviation medicine training can be added to the education curriculum of medicine [37,45]. There are a few up-and-coming technologies that are useful for modern telemedicine. For example, Wi-Fi has been gradually used in airplanes [46,47]. The airline company that provided the IME data in our study has already achieved full coverage of Wi-Fi on wide-body aircraft, providing technical support for remote sound and image transmissions, and wearable devices are already being used on board the company’s aircraft for vital sign transmission.

### Limitations

Our study has several major limitations that must be noted. First, the IME data we used in this analysis were from one of the leading airline companies in mainland China, which might not represent the whole population. However, this company was one of the three leading airline companies and covered a substantially large proportion of the total flow of air travel. Second, the collected data was incomplete. For example, the outcomes for some patients after leaving the aircraft were missing, and the medication and treatment records were incomplete. The IME reporting process in China is currently not standardized, and the circumstances surrounding reporting differ across airlines. Since many IME reports are completed by nonmedical flight attendants, some information may be erroneous or missing, such as the passenger’s symptoms and the emergency procedures performed. Moreover, tracking the health outcomes of passengers off the plane is not practical because of flight mobility and missing persons after disembarkation. Due to the relatively low frequency of IMEs, along with crew workloads, certain small instances may not be adequately recorded or included in the official IME reporting system. Currently, Chinese airlines rely mostly on internal reporting and records, which makes the entire assessment and improvement of IMEs difficult. Therefore, it is crucial to enhance the electronic IME reporting system to ensure the comprehensive documentation of all IMEs. Third, the medical categories for IMEs were based on descriptions of the passenger’s primary symptoms in the IME record and not on formal diagnoses. However, 3 doctors were invited to individually evaluate the symptoms to improve the accuracy of the medical categories.

### Conclusion

Based on our study, we found that the incidence of IMEs and IME-related deaths remained rare in China. There is a temporal trend in IME occurrences, with more incidences occurring at night and in the winter. Narrow-body aircraft have a lower incidence rate of IMEs but have a higher diversion rate. Cardiac symptoms, seizures, and syncope or presyncope are the three leading IMEs. Companies that provide airline passenger transportation should provide more ground-to-air medical support for patient management and aircraft decision-making. Since IME records are often incomplete, our study highlights the importance of a unified reporting system for IMEs globally.

## Supplementary material

10.2196/63557 Multimedia Appendix 1 Supplementary analyses and materials.

## Abbreviations

IME: in-flight medical emergency
OR: odds ratio
