# Supplementary material for: Characteristics of In-Flight Medical Emergencies on a Commercial Airline in Mainland China: Retrospective Study
Source: JMIR Public Health Surveill. 2024 Dec 19;10:e63557. doi: 10.2196/63557 (PMC11671026; doi:10.2196/63557)
Supplement: Multimedia Appendix 1 [file publichealth-v10-e63557-s001.docx]

content.

[Figure S1 Management of in-flight medical emergencies for the common diseases or symptoms 2](#_Toc18575)

[Table S1 Characteristics of in-flight emergency by outcome of passenger 3](#_Toc4683)

[Table S2 Multivariable analysis of factors associated with aircraft diversion 4](#_Toc5006)

[Table S3 Emergency medical incident report form. 5](#_Toc24913)

[Table S4 Contents of emergency medical kits. 7](#_Toc31889)


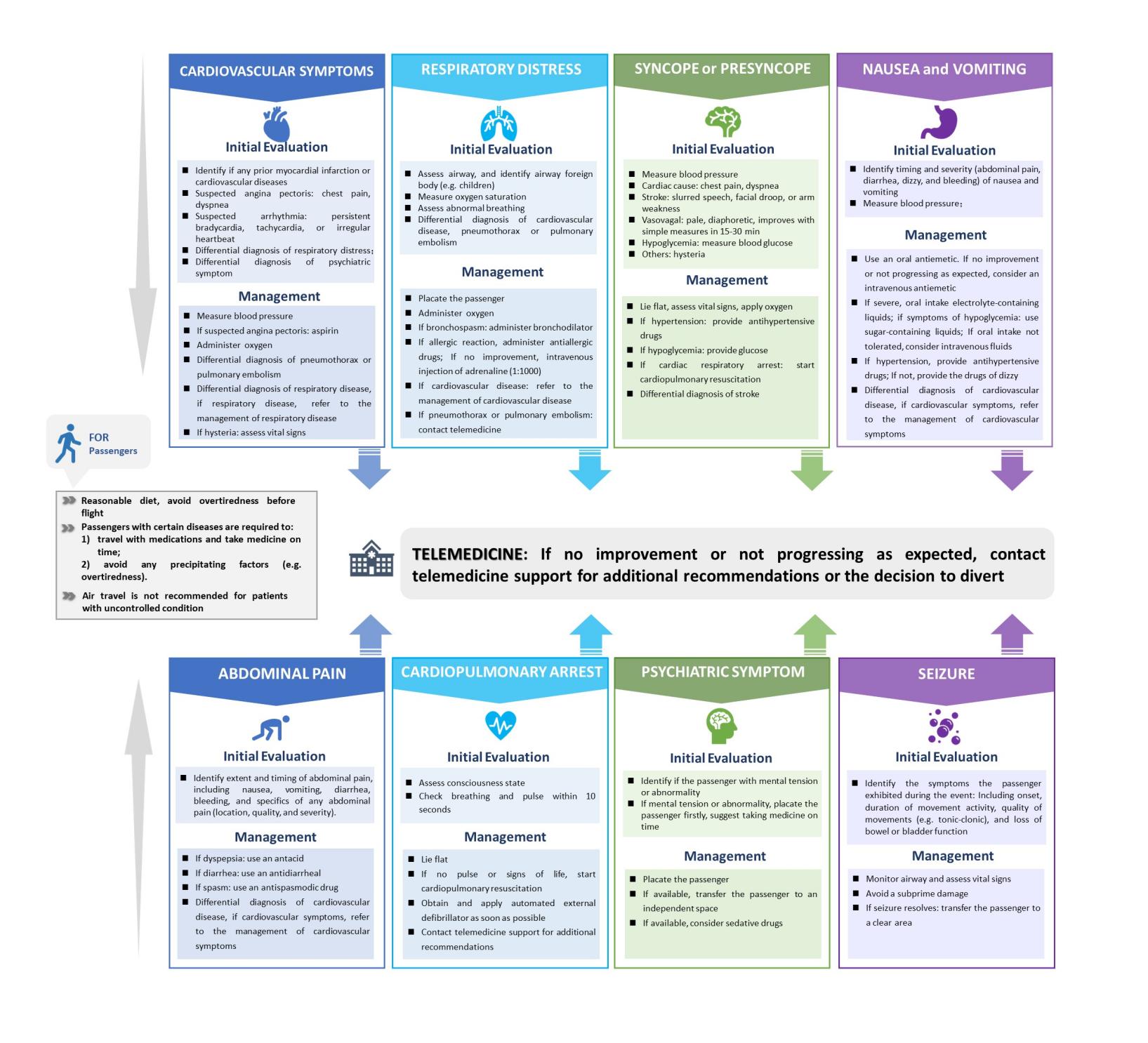


# Figure S1 Management of in-flight medical emergencies for the common diseases or symptoms

# Table S1 Characteristics of in-flight emergency by outcome of passenger

| **Variables** | **All**  **N (%)** | **Survived**  **n (%)** | **Died**  **n (%)** | **Unknown**  **n (%)** | **P value** |
| --- | --- | --- | --- | --- | --- |
| Total (n) | 199 |  |  |  | <0.001 |
| Age groups (years, %) | |  |  |  |  |
| <18 | 13(6.5) | 11(11.6) | 1(4.2) | 3(1.3) |  |
| 18-34 | 35(17.6) | 23(24.2) | 2(8.3) | 10(12.5) |  |
| 35-49 | 24(12.1) | 12(12.6) | 2(8.3) | 10(12.5) |  |
| 50-64 | 41(20.6) | 22(23.2) | 6(25.0) | 13(16.3) |  |
| ≥65 | 23(11.6) | 8(8.4) | 9(37.5) | 6(7.5) |  |
| Unspecified | 63(31.7) | 19(20.0) | 4(16.7) | 40(50.0) |  |
| Gender (%) |  |  |  |  | <0.001 |
| Female | 71(35.7) | 46(48.4) | 6(25.0) | 19(23.8) |  |
| Male | 82(41.2) | 38(40.0) | 15(62.5) | 29(36.2) |  |
| Unspecified | 46(23.1) | 11(11.6) | 3(12.5) | 32(40.0) |  |
| Type of aircraft (%) | |  |  |  | 0.036 |
| Wide-body plane | 52(26.1) | 28(29.5) | 10(41.7) | 14(17.5) |  |
| Narrow-body plane | 147(73.9) | 67(70.5) | 14(58.3) | 66(82.5) |  |
| Flight distance (%) | |  |  |  | 0.046 |
| <1000 | 16(8.0) | 11(11.6) | 1(4.2) | 4(5.0) |  |
| 1000-1999 | 91(45.7) | 41(43.2) | 8(33.3) | 42(52.5) |  |
| 2000-3999 | 49(24.6) | 19(20.0) | 6(25.0) | 24(30.0) |  |
| ≥4000 | 43(21.6) | 24(25.3) | 9(37.5) | 10(12.5) |  |
| Volunteer provider of medical assistance (%) | | | |  | <0.001 |
| No | 147(73.9) | 66(69.5) | 10(41.7) | 71(88.8) |  |
| Yes | 52(26.1) | 29(30.5) | 14(58.3) | 9(11.2) |  |
| Aircraft diversion (%) | 136 (68.3) | 48(50.5) | 11(45.8) | 77(96.2) |  |
| AED use* (%) | 6(33.3) | 1(1.1) | 5(20.8) | 0(0.0) | <0.001 |
| Medical history (%) | |  |  |  | 0.002 |
| No | 42(21.1) | 29(30.5) | 2(8.3) | 11(13.7) |  |
| Yes | 61(30.7) | 32(33.7) | 10(41.7) | 19(23.8) |  |
| Unspecified | 96(48.2) | 34(35.8) | 12(50.0) | 50(62.5) |  |
| Season (%) |  |  |  |  | 0.006 |
| Jan. | 23(11.56) | 8(8.42) | 3(12.5) | 12(15) |  |
| Feb. | 13(6.53) | 5(5.26) | 0(0) | 8(10) |  |
| Mar. | 19(9.55) | 6(6.32) | 2(8.33) | 11(13.75) |  |
| Apr. | 19(9.55) | 5(5.26) | 5(20.83) | 9(11.25) |  |
| May | 8(4.02) | 2(2.11) | 3(12.5) | 3(3.75) |  |
| Jun. | 17(8.54) | 9(9.47) | 1(4.17) | 7(8.75) |  |
| Jul. | 8(4.02) | 3(3.16) | 2(8.33) | 3(3.75) |  |
| Aug. | 7(3.52) | 3(3.16) | 3(12.5) | 1(1.25) |  |
| Sep. | 22(11.06) | 15(15.79) | 1(4.17) | 6(7.5) |  |
| Oct. | 19(9.55) | 15(15.79) | 1(4.17) | 3(3.75) |  |
| Nov. | 24(12.06) | 13(13.68) | 0(0) | 11(13.75) |  |
| Dec. | 20(10.05) | 11(11.58) | 3(12.5) | 6(7.5) |  |
| Year(%) |  |  |  |  | <0.001 |
| 2018 | 58(29.2) | 10(10.5) | 4(16.7) | 44(55.0) |  |
| 2019 | 74(37.2) | 62(65.3) | 12(50.0) | 0(0.0) |  |
| 2020 | 21(10.6) | 3(3.2) | 1(4.2) | 17(21.2) |  |
| 2021 | 26(13.1) | 3(3.2) | 4(16.7) | 19(23.8) |  |
| 2022 | 20(10.0) | 17(17.9) | 3(12.5) | 0(0.0) |  |

Note: AED, automated external defibrillator. *Only calculated among cardiopulmonary arrest cases

# Table S2 Multivariable analysis of factors associated with aircraft diversion

| Variable | aOR(95%CI) | P-value |
| --- | --- | --- |
| Age groups |  |  |
| <18 | 1.00 |  |
| 18-34 | 0.47(0.05-4.58) | 0.519 |
| 35-49 | 0.53(0.05-5.83) | 0.601 |
| 50-64 | 0.77(0.08-7.31) | 0.822 |
| ≥65 | 0.10(0.01-1.30) | 0.078 |
| Gender |  |  |
| Female | 1.00 |  |
| Male | 1.50(0.54-4.19) | 0.441 |
| Type of aircraft |  |  |
| Wide-body plane | 1.00 |  |
| Narrow-body plane | 7.75(0.76-79.13) | 0.044 |
| Flight distance |  |  |
| <1000 | 1.00 |  |
| 1000-1999 | 4.74(1.31-17.08) | 0.017 |
| 2000-3999 | 2.43(0.60-9.75) | 0.212 |
| ≥4000 | 16.40(1.78-151.29) | 0.014 |
| Medical problems |  |  |
| Hysteria, agitation or psychiatric symptoms | 1.00 |  |
| Cardiac symptoms | 5.09(0.28-93.55) | 0.273 |
| Seizures | 0.24(0.03-2.13) | 0.200 |
| Syncope or presyncope | 0.64(0.05-8.24) | 0.730 |
| Abdominal pain, nausea, or vomiting | 0.33(0.03-3.93) | 0.383 |
| Cardiopulmonary arrest | 0.26(0.02-3.84) | 0.330 |
| Respiratory symptoms | 0.36(0.03-5.03) | 0.445 |
| Others | 0.28(0.03-2.36) | 0.242 |
| Unknown | 0.17(0.01-1.94) | 0.152 |
| Season |  |  |
| March-May | 23.21(3.75-143.43) | 0.001 |
| June-August | 2.75(0.56-13.45) | 0.211 |
| September-November | 1.00 |  |
| December-February | 12.70(3.09-52.23) | <0.001 |

# Table S3 Emergency medical incident report form.

**Emergency Medical Incident Report Form**

**Passenger’s basic information**

| Name |  | Gender |  | Age |  |
| --- | --- | --- | --- | --- | --- |
| Flight |  | Seat |  | Nationality |  |
| Date |  | Alternate |  | Passport No. |  |
| Telephone |  | Address |  | | |

**Major Discomfort**

🞏(1) Coma 🞏(2) Dyspnea 🞏(3) Chest pain 🞎(4) Palpitation 🞏(5) Abdominal pain

🞏(6) Fever 🞏(7) Cough and expectoration 🞎(8) Diarrhea 🞏(9) Nausea and vomiting

🞏(10) Twitch 🞏(11) Earache 🞏(12) Headache 🞎(13) Abdominal discomfort

🞏(14) Hemorrhage 🞏(15) Dizziness 🞏(16) Insanity 🞏(17) Rash 🞏(18) Trauma

🞎(19) Arm or leg pain 🞏(20) Pregnancy symptoms 🞏(21) Pediatric symptoms (Inhalation of foreign objects, allergy or trauma)

🞏(22) Others: __________________________________________________

**Description**

| **NO.** | **Starting Time** | **Duration** | **Phase** | **Additional Information** |
| --- | --- | --- | --- | --- |
|  |  |  |  |  |
|  |  |  |  |  |
|  |  |  |  |  |

**Interpretation:** 1. ”NO.” refers to the ordinal number of the selected discomfort; 2. “Starting Time” refers to when the discomfort started; 3. “Duration” refers to the duration of discomfort; 4. “Phase”: Such as taxiing, takeoff, cruise or landing; 5. “Additional Information”: For instance, accompanying symptoms, techniques for alleviation, nature of pain and so forth.

**Types, names, and doses of medications taken before boarding the flight.**

| 🞏Sleeping pills |  | 🞏Hypotensive medicine |  | 🞏Diabetes medication |  |
| --- | --- | --- | --- | --- | --- |
| 🞏Antiallergics |  | 🞏Antiepileptics |  | 🞏Anticoagulants |  |
| 🞏Other |  | | | | |

Page 1/2

**Past medical history**

🞏 1: Hypertension 🞏 2: Diabetes 🞏 3: Coronary disease 🞏 4: Cerebrovascular accident

🞏5: Chronic hepatitis 🞎6: Chronic pulmonary diseases 🞏 7: Epilepsy 🞏 8: Syncope

🞎9: Ulcer/chronic inflammation of digestive tract 🞏 10: Food Allergy

🞏11: Arrhythmia 🞏12: Rheumatoid autoimmune disease 🞎13: Tumor maintenance therapy

🞏14: Surgical history within 1 month before boarding 🞏 15: Drug Allergy

🞏16: Otolaryngological diseases 🞏 17: Psychiatric illness 🞏 18: Urinary system calculi

🞏19: Eye disease 🞏 20:Other:

***Additional Information*** (Such as the duration of medical history, specific allergic reactions, and surgical methods.):

**Basic vital signs**

|  | **Data** | **Record time** |
| --- | --- | --- |
| Heart rate |  |  |
| Respiratory |  |  |
| BP（mmHg） |  |  |
| Glucose(mmol/L) |  |  |
| T(℃) |  |  |
| SaO2（%） |  |  |

**Measures Taken:**

**Outcomes:**

**Consumed Items:**

| Witness | Address/Telephone | Nationality & Passport NO. | SEAT | Signature |
| --- | --- | --- | --- | --- |
|  |  |  |  |  |
|  |  |  |  |  |
| Processor | Address | Telephone | | Signature |
|  |  |  | |  |
|  |  |  | |  |

**Date: Signature of the purser**

Page 2/2

# Table S4 Contents of emergency medical kits.

|  | **Equipment** | **Medications** |
| --- | --- | --- |
| CAAC-mandated emergency medical kits（2011） | Arterial tourniquet (rubber, pneumatic and cloth tourniquets)  Bandage  Dressings  Instructions on kit use  Medical rubber gloves(sterilized)  Medical scissors  Medical tape  One-way valve mouth-to-mouth resuscitation mask  Oropharyngeal airway(tube)  Splints  Syringes and needles  Triangular bandage | Nitroglycerin tablets,0.4mg  Normal saline  Skin disinfectants (non-alcohols such as iodophor and chlorhexidine) |
| Items recommended for normal medical kits | CPR mask  Glucometer  Intravenous administration set(indwelling needles)  Medical penlight  Prefilled syringes  Pulse oximeter  Sphygmomanometer  Stethoscope  Thermometer | Antacids (eg, omeprazole enteric-coated capsules,20mg and hydrotalcite tablets,0.5g)  Antihistamine tablets (eg. Loratadine tablets,10mg）  Aspirin tablets,325mg  Belladonna tablets,10mg  Bronchodilator, inhaled (eg. salmeteroticasone powder inhalant aerosol,50ug:100ug)  Clonidine hydrochloride tablets，0.1mg  Ibuprofen sustained-released capsule,0.3g |
| Items recommended for enhanced medical kits | AED (Automated External Defibrillator)  POCT (Point-of-care testing, especially for cardiac biomarkers, blood gas analysis, and electrolytes respectively) | Dexamethasone, injectable (eg.Dexamethasone Sodium Phosphate Injection,1ml:5mg)  Dextrose, 50%/50 cc injectable, (single dose ampule or equivalent)  Epinephrine 1:1000, 1 cc, injectable, (single dose ampule or equivalent)  Lidocaine, 5 cc, 20 mg/ml, injectable (single dose ampule or equivalent)  Low molecular weight heparin sodium injection,0.4ml,4000 I.U.aXa |

The equipment and drugs in the medical kit should be able to be stored at room temperature and checked regularly to ensure that they are within their expiration date. It is also important to regularly update the medical kit

CAAC: Civil Aviation Administration of China
